# Correction of Iatrogenic Facial Asymmetry Caused by Botulinum Toxin Using Contralateral Injection: A Case Report

**DOI:** 10.1093/asjof/ojag051

**Published:** 2026-03-20

**Authors:** Muhammad Rayyan Bilal, Rana Hassan Javaid, Afsheen Bilal

## Abstract

Facial symmetry is a key determinant of aesthetic harmony and psychosocial well-being. Iatrogenic asymmetry is a recognized complication of botulinum toxin injections and may cause both functional and emotional distress. Contralateral botulinum toxin adjustment is an accepted corrective strategy, but few case reports have documented its use for toxin-induced asymmetry with photographic evidence. A 44-year-old woman developed facial asymmetry after cosmetic botulinum toxin injections. She presented with upward deviation of the left oral commissure and midface both at rest and during speech, causing psychosocial discomfort. Dynamic assessment showed relative overactivity of the left facial muscles compared with the weakened right side. Targeted contralateral injections of botulinum toxin type A were delivered into the left zygomaticus minor, depressor anguli oris, and mentalis muscles (14 units). At the 2-week follow-up, the patient showed substantial restoration of facial balance with improved commissure position and symmetry. Posttreatment photographs confirmed correction. No adverse events were observed. Contralateral botulinum toxin injection is a possible minimally invasive method for correcting toxin-induced asymmetry.

**Level of Evidence: 5 (Therapeutic)**  
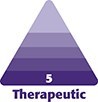

Facial symmetry strongly influences perceptions of beauty, health, and social interaction. Studies consistently demonstrate that symmetrical faces are regarded as more attractive and healthier across cultures.^1,2^ With the increasing use of aesthetic procedures, clinicians are more frequently encountering iatrogenic asymmetry, often related to filler maldistribution, toxin diffusion, injection errors, or procedure-induced nerve dysfunction. These complications can cause both functional and emotional distress for patients.

Traditional corrective strategies, including filler dissolution, surgical revision, or observation, have important limitations and often involve downtime. Botulinum toxin type A (BoNTA) offers a targeted and reversible approach that can restore balance by modulating relative overactivity on the contralateral side. Its role in facial palsy, synkinesis, and related asymmetries has been well described.^3-5^ Guidelines emphasize conservative dosing, thorough pretreatment assessment, and careful planning.^5^

Here, we describe a case of contralateral BoNTA injection used to correct toxin-induced facial asymmetry, resulting in rapid improvement and meaningful psychosocial benefit.

## CASE PRESENTATION

A 44-year-old woman presented with facial asymmetry 3 weeks after receiving cosmetic botulinum toxin treatment for facial wrinkles. She reported that her smile appeared “uneven,” with diminished balance and self-confidence. On clinical examination, the right side of the face appeared flattened and flaccid, whereas the left oral commissure was elevated at rest and during speech, producing noticeable asymmetry (Figure 1A). Dynamic assessment demonstrated relative overaction of the left facial muscles compared with contralateral weakness, accentuating lip deviation and imbalance (Figure 1B). Symptoms became apparent within days of the initial injection and persisted for ∼3 weeks before presentation.

**Figure 1.**
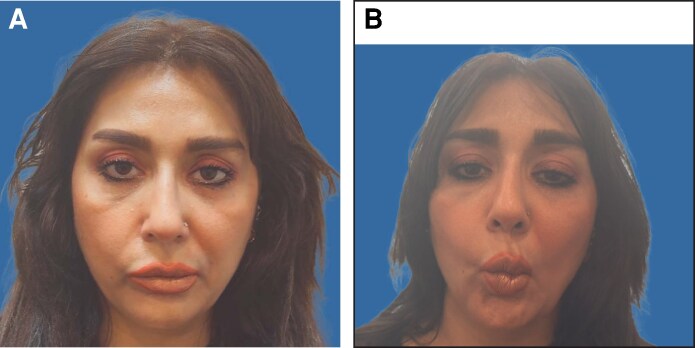
(A) Pretreatment photograph showing right-sided facial asymmetry following previous botulinum toxin injection. Note right-sided flattening with relative upward pull of the left oral commissure. (B) Additional pretreatment dynamic puckering view highlighting overactivity of the left perioral muscles compared with contralateral weakness, accentuating lip deviation. Patient is a 44-year-old female.

After thorough counseling and informed consent, contralateral BoNTA was used to correct the iatrogenic facial asymmetry following previous unilateral injection. The toxin was reconstituted in 100 units/2.5 mL saline according to standard clinical practice. Injection sites and doses were selected based on detailed anatomical assessment and the degree of contralateral muscle overactivity contributing to asymmetry. Using a 30-gauge needle, 6 units were injected into the left zygomaticus minor muscle, divided as 2 units at the upper insertion and 4 units along the muscle belly over the zygomatic region. Additionally, 2 units were administered into the left depressor anguli oris muscle and 6 units into the mentalis muscle. Conservative dosing was intentionally chosen based on clinical judgment and visual assessment of muscle activity to minimize the risk of further asymmetry or overcorrection. A follow-up assessment was done at 2 weeks.

Although mild upper-facial differences were visible, they were not clinically relevant to the patient's main concern of left-sided oral commissure elevation. Mechanistically, unintended diffusion from more lateral and inferior crow's feet injection points on the right, together with relative underdosing of the left depressor anguli oris and mentalis, most plausibly produced the observed pattern (Figure 2).

**Figure 2.**
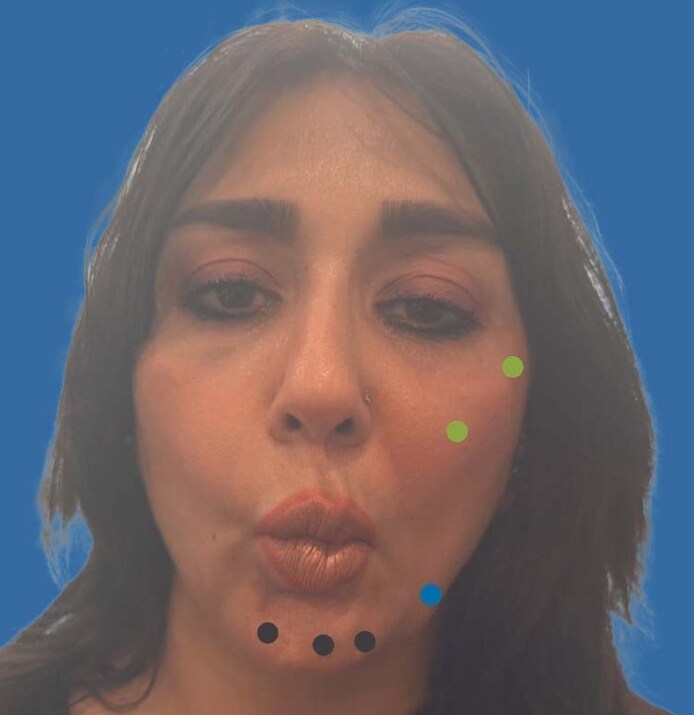
Marked injection points for contralateral botulinum toxin placement on the left side: green (1, 2) = zygomaticus minor (2 units + 4 units); blue (3) = depressor anguli oris (2 units); black (4-6) = mentalis (6 units total). Patient is a 44-year-old female.

At the 2-week follow-up, the patient demonstrated marked improvement in commissure position and overall facial symmetry (Figure 3). She reported high satisfaction and relief of psychosocial distress. No adverse effects were observed.

**Figure 3.**
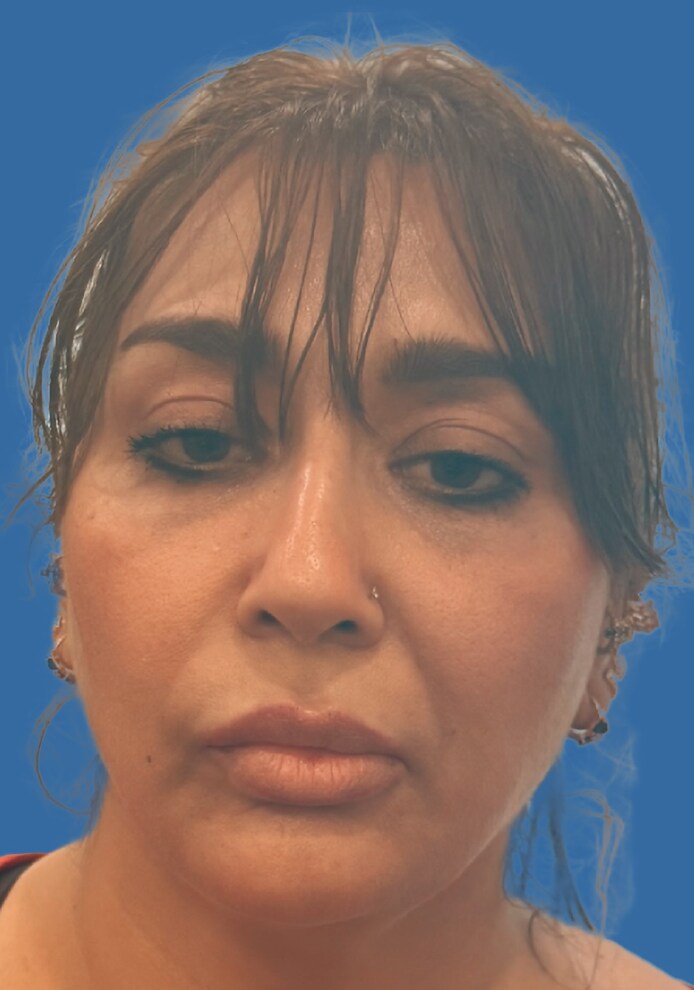
Posttreatment photograph at 2 weeks after contralateral botulinum toxin injection, showing restoration of commissure balance and improved facial symmetry. Patient is a 44-year-old female.

Written informed consent for publication of clinical details and images was obtained from the patient.

## DISCUSSION

BoNTA has been widely applied in managing facial palsy by modulating contralateral activity to restore dynamic balance. Kim et al reported favorable outcomes in acute facial paralysis by injecting the unaffected side, whereas Choi et al demonstrated similar benefits in synkinesis management.^3,4^ Heydenrych emphasized careful assessment and conservative dosing, and Cooper's systematic review confirmed overall efficacy of BoNTA in facial palsy contexts.^5,6^ Additional reports have demonstrated improvement in lower facial symmetry and contour through targeted toxin modulation.^7,8^

The advantages of contralateral injection include its minimally invasive nature, negligible downtime, and ability to achieve natural improvement. Patients particularly value the immediacy of benefit compared with surgical or expectant management. This case shows how the same principles can be applied to iatrogenic toxin-induced imbalance. Although familiar in practice, few case reports have documented this approach with photographic evidence, which adds practical and educational value for clinicians.

Subtle preexisting facial asymmetry, such as minor commissure height difference or cheek fullness variation, may contribute to the overall imbalance and may likely be accentuated by toxin diffusion or dosing variation. The selected doses were intentionally conservative and tailored to the observed pattern of muscle overactivity. Fractionated dosing of the zygomaticus minor allowed graded weakening while preserving smile dynamics, whereas lower doses were used for the depressor anguli oris to minimize the risk of oral incompetence. Mentalis dosing was chosen to correct chin imbalance without impairing speech or mastication. This individualized approach aimed to restore symmetry while avoiding overcorrection.

Given the clear relative hyperactivity on the untreated side, contralateral injection was performed with conservative dosing and precise anatomical targeting to restore balance rather than induce new weakness. The patient was counseled regarding potential transient functional effects, none of which were observed during follow-up.

Successful correction requires accurate diagnosis, detailed anatomical mapping, and precise injection to avoid undesired outcomes. The temporary effect of BoNTA is a limitation, as repeated treatments may be required. In our patient, however, the complication itself was temporary, caused by previous toxin exposure, and was corrected within days by contralateral dosing.

This report has several limitations. Exact doses and injection sites from the initial botulinum toxin treatment were unavailable. Only static posttreatment photographs were available, and dynamic images such as pursed lips were not obtained. In addition, photographic quality was limited to available clinical records. Finally, this report describes a single patient, which limits generalizability. Despite these constraints, the case highlights an important clinical lesson: contralateral BoNTA injection can provide an effective and reversible strategy for restoring facial symmetry following toxin-induced complications.

Contralateral botulinum toxin injection may be considered when clear relative hyperactivity is present and spontaneous improvement is unlikely, whereas observation remains appropriate for mild or early asymmetry given the temporary nature of toxin effects. Progressive weakness, sensory deficits, or atypical patterns should prompt neurological evaluation rather than aesthetic correction alone. Patients should be counseled that both the complication and its correction are temporary and may require reassessment.

## CONCLUSIONS

Contralateral botulinum toxin injection represents a possible minimally invasive approach for correcting botulinum toxin–induced facial asymmetry in selected cases.
